# *Hibecovirus* (genus *Betacoronavirus*) infection linked to gut microbial dysbiosis in bats

**DOI:** 10.1093/ismeco/ycae154

**Published:** 2024-12-16

**Authors:** Dominik W Melville, Magdalena Meyer, Alice Risely, Kerstin Wilhelm, Heather J Baldwin, Ebenezer K Badu, Evans Ewald Nkrumah, Samuel Kingsley Oppong, Nina Schwensow, Marco Tschapka, Peter Vallo, Victor M Corman, Christian Drosten, Simone Sommer

**Affiliations:** Institute of Evolutionary Ecology and Conservation Genomics, Ulm University, Ulm, BW 89081, Germany; Institute of Evolutionary Ecology and Conservation Genomics, Ulm University, Ulm, BW 89081, Germany; Institute of Evolutionary Ecology and Conservation Genomics, Ulm University, Ulm, BW 89081, Germany; School of Science, Engineering, and the Environment, Salford University, Salford M5 4NT, UK; Institute of Evolutionary Ecology and Conservation Genomics, Ulm University, Ulm, BW 89081, Germany; Institute of Evolutionary Ecology and Conservation Genomics, Ulm University, Ulm, BW 89081, Germany; School of Natural Sciences, Macquarie University, Sydney, New South Wales 2113, Australia; Department of Wildlife and Range Management, Kwame Nkrumah University of Science and Technology, AK-385-1973, Kumasi, Ghana; Department of Wildlife and Range Management, Kwame Nkrumah University of Science and Technology, AK-385-1973, Kumasi, Ghana; Department of Wildlife and Range Management, Kwame Nkrumah University of Science and Technology, AK-385-1973, Kumasi, Ghana; Institute of Evolutionary Ecology and Conservation Genomics, Ulm University, Ulm, BW 89081, Germany; Institute of Evolutionary Ecology and Conservation Genomics, Ulm University, Ulm, BW 89081, Germany; Institute of Evolutionary Ecology and Conservation Genomics, Ulm University, Ulm, BW 89081, Germany; Institute of Vertebrate Biology, Czech Academy of Sciences, Brno 675 02, Czech Republic; German Centre for Infection Research (DZIF) and Charité—Universitätsmedizin Berlin Institute of Virology, Berlin 10117, Germany; German Centre for Infection Research (DZIF) and Charité—Universitätsmedizin Berlin Institute of Virology, Berlin 10117, Germany; Institute of Evolutionary Ecology and Conservation Genomics, Ulm University, Ulm, BW 89081, Germany

**Keywords:** coronavirus, microbiome, Chiroptera, Anna Karenina principle, Ghana

## Abstract

Little is known about how zoonotic virus infections manifest in wildlife reservoirs. However, a common health consequence of enteric virus infections is gastrointestinal diseases following a shift in gut microbial composition. The sub-Saharan hipposiderid bat complex has recently emerged to host at least three coronaviruses (CoVs), with *Hipposideros caffer D* appearing particularly susceptible to *Hibecovirus* CoV-2B infection. In this study, we complement body condition and infection status data with information about the gut microbial community to understand the health impact of CoV infections in a wild bat population. Of the three CoVs, only infections with the distantly SARS-related *Hibecovirus* CoV-2B were associated with lower body condition and altered the gut microbial diversity and composition. The gut microbial community of infected bats became progressively less diverse and more dissimilar with infection intensity, arguing for dysbiosis as per the Anna Karenina principle. Putatively beneficial bacteria, such as *Alistipes* and *Christensenella*, decreased with infection intensity, while potentially pathogenic bacteria, namely *Mycoplasma* and *Staphylococcus*, increased. Infections with enterically replicating viruses may therefore cause changes in body condition and gut dysbiosis with potential negative health consequences even in virus reservoirs. We argue that high-resolution data on multiple health markers, ideally including microbiome information, will provide a more nuanced picture of bat disease ecology.

## Introduction

A stable and diverse gut microbiome is widely understood to indicate host health [1-3] and determine host fitness [4-6]. Perturbations can lead to idiosyncratic and unstable configurations of the gut microbial community (i.e., dysbiosis following the Anna Karenina principle: ‘all healthy microbiomes are similar; each dysbiotic microbiome is dysbiotic in its own way’) [2, 7], and impair metabolic and immunological functions provided by the gut microbiota [8-10]. A possible though not immediately visible health consequence of a virus infection is the reshuffling of the host-associated gut microbiota and gastrointestinal disease [11-13]. However, compared with humans or mice, we know very little about how the microbiota of wildlife responds to viral challenges [14-17], and even less is known about how the microbiota of pathogen reservoir hosts copes with virus infections.

Bats are among the best studied virus reservoirs, albeit rarely showing signs of disease [18-23]. Compared with the physiological and behavioral responses to infections (e.g., [24, 25]), our knowledge about how the gut microbial community of bats responds to infections is limited [26]. Their short gut transit times [27, 28] and their supposedly low level of phylosymbiosis [29, 30] even has brought into question whether bats rely on their gut microbiota [31, 32]. However, the enlarged digestive area by villi offers niches for microbiota in bats [33], and bat gut microbes are enriched in genes supporting nutrient acquisition [34-38] and immunity [39, 40]. Moreover, Liu *et al.* (2022) attests to a potential role of bat gut microbiota in pathogen tolerance. The researchers transplanted the gut microbiota from wild Great Himalayan roundleaf bats (*Hipposideros armiger*) into antibiotic-treated mice, and found a rapid engagement with cytotoxic innate and adaptive immune pathways, culminating in tolerating a challenge with the influenza virus H1N1 [40]. By contrast, the microbiota shifted toward a bacterial community dominated by pathogenic taxa in wild Jamaican fruit bats (*Artibeus jamaicensis*) infected with an enteric Astrovirus [41]. To date, the work by Wasimuddin and colleagues (2019) remains the only example to document gut microbial dysbiosis following a virus infection in wild bats, although this shift had no repercussions on host body condition. This paucity of studies investigating bat-microbiome-virus interactions greatly limits our understanding of the role of gut microbiota in resisting, tolerating and clearing virus infections.

Coronaviruses (CoVs) count among the most diverse viral families discovered in bats globally with at least five now known to have crossed species boundaries and spilled over into humans causing mild to severe respiratory tract infections [21, 42, 43]. In humans and non-bat animal hosts, some of these viruses alter the composition of the gut microbial community [44-47], modulating the host’s immunological response to the infection [48]. Given that CoVs replicate enterically in bats [49], CoV infections could also feasibly alter their gut microbial community in bats. Cave-dwelling, sub-Saharan roundleaf bats (genus: *Hipposideros*), which form a diverse species complex [50], have recently emerged as ancestral hosts to the *Alphacoronavirus* HCoV-229E, which causes mild cold symptoms in humans [43]. Furthermore, hipposiderids host at least two *Betacoronaviruses*, provisionally termed CoV-2B and CoV-2Bbasal [43, 51, 52]. Interestingly, the viruses show uneven infection patterns among the co-inhabiting *Hipposideros* species: CoV-229E is most prevalent among *Hipposideros (H.) caffer C*, whereas *H. caffer D* is likely the main reservoir host to both *Betacoronaviruses* [51]. Moreover, immune genes involved in adaptive immunity were associated with susceptibility of *H. caffer D* to either SARS-related *Betacoronavirus* [53]. Yet, knowledge about whether the body condition and gut microbiota of such reservoir species change with CoV infection is lacking.

In this article, we explore the link between two host health markers, i.e., body condition and gut microbial community, and infections with either of three distinct CoVs. First, we assessed whether the body condition of Sundevall’s roundleaf bat (*H. caffer D*) infected with either of three distinct CoVs (i.e., the *Hibecoviruses* CoV-2B and CoV-2Bbasal and the *Duvinacovirus* CoV-229E) declined. A change in body condition is an important health indicator in bats [54], although rarely reported so far (but see: [19, 23]). Second, we test whether the diversity and composition of the gut microbiota found in uninfected bats differed from that of bats positive for either of the three CoVs, and whether the change correlates with infection intensity. Because *H. caffer D* was suggested to be the main reservoir to both SARS-related *Betacoronaviruses* [51, 53], we hypothesize that only these infections affect body condition and microbiota. The hypothesis-driven approach and virus screening down to species level sets our work apart from previous research [41]. In addition, linking dysbiosis with infection intensity adds a quantifiable dimension to the Anna Karenina principle [7]. We leverage virus infection and body condition information from 591 adult bats collected from five cave sites in Ghana between 2010 and 2012 and high quality 16S ribosomal ribonucleic acid (rRNA) microbiome data from a subsample of 218 bats (n_uninfected_ = 46; n_CoV2B_ = 41; n_CoV2Bbasal_ = 70; n_CoV229E_ = 61). Our results demonstrate that enterically replicating viruses can alter the body condition and gut microbial community even in virus reservoirs.

## Material and methods

### Sampling design

Sampling protocols and methods were described in detail elsewhere [51]. Briefly, bats were captured at five different cave sites in central Ghana, West Africa: Buoyem 1 (N7°72′35.833” W1°98′79.167), Buoyem 2 (N7°72′38.056” W1°99′26.389), Forikrom (N7°58′97.5” W1°87′30.299), Kwamang 1 (N6°58′0.001” W1°16′0.001), and Kwamang 2 (N7°43′24.899” W1°59′16.501) in more than 12 sampling events spread evenly over the span of 2 years (September 2010–August 2012). Each sampling event consisted of mist net trapping at the cave entrance 1 hour after dusk until dawn for two non-consecutive nights. *H. caffer D* was identified molecularly by sequencing the *cytb* gene from 2 mm wing punches collected at sampling [50, 51, 53]. The species nomenclature of the *H. caffer* complex remains unresolved throughout the Afrotropics, but here we use *H. caffer D*, as an interim species name [50, 51]. Additionally, fecal pellets were collected from bats held individually in clean bags until defecation, and stored in RNAlater (Life Technologies, USA) at −80°C for subsequent virus and microbiome screening [51]. Body mass (g) and forearm length (mm) were taken and used to calculate the body condition index (as body mass/forearm length) for non-pregnant adults. This relationship between body mass and forearm length can be used as a proxy to estimate the impact of infections generally [54] and in bats in particular [23]. A higher body condition index implies bats have more fat reserves, possibly indicating superior health [26]. The Wildlife Division of the Forestry Commission of the Ministry of Lands, Forestry and Mines granted the research (A04957) and ethics permit (CHRPE49/09/CITES).

### Virus screening

Approximately 20 mg of the fecal material was suspended in 500 μl RNAlater stabilizing solution (QIAGEN, Hilden, Germany) and homogenized via vortexing before extracting and purifying viral RNA with the MagNa Pure 96 system (Roche, Penzberg, Germany) [43, 55]. Elution volumes were set at 100 μl. Subsequently, a real-time reverse transcription-polymerase chain reaction (rt-PCR) assay was designed to detect several *Alphacoronaviruses* and *Betacoronaviruses*, as described previously [43, 51, 52, 56]. In each PCR run, *in vitro* transcribed and photometrically quantified RNAs, generated from TA-cloned periamplicons using the T7-driven MEGAscript (Life Technologies, Heidelberg, Germany), were used as positive controls, and calibrators to ensure run-to-run consistency [51, 57]. A total of four CoVs were described: A MERS-related *Betacoronavirus* termed 2C exclusively found in samples originating from *Nycteris macrotis* [51, 56], the *Alphacoronavirus* CoV-229E-like as the closest known ancestral form to the HCoV-229E [43], and two distantly SARS-related *Betacoronaviruses*, named CoV-2B and CoV-2Bbasal [51, 52]. Genome-level analysis according to recent taxonomical amendments assigned both *Betacoronaviruses* to the subgenus *Hibecovirus,* which was previously included in the genus *Sarbecovirus*, and the *Alphacoronavirus* to the subgenus *Duvinacovirus* (author C.D., own unpublished observations). Our focal species *H. caffer D* hosts both *Hibecoviruses* and the *Duvinacovirus* CoV-229E-like [51]. Notably, *H. caffer D* is thought to be particularly susceptible to infections with the *Hibecovirus* 2B [51, 53], which hints at the species’ role as the main reservoir to this virus. After viral screening, we recorded the infection status as category (i.e., positive for CoV-229E-like, CoV-2B, or CoV-2Bbasal) and infection intensity estimated based on cycle threshold (CT) value for each sample. Low CT-values imply a more acute infection, and were previously found in *H. caffer D* infected with *Hibecovirus* CoV-2B compared with closely related hipposiderids [51].

### 16S ribosomal ribonucleic acid sequencing and bioinformatics

A subset of 221 fecal samples representing each infection status with roughly similar sample sizes was chosen for 16S rRNA sequencing. Because of the confounding effect of co-infection [58], samples from bats with more than one virus infection were not included. Bacterial deoxyribonucleic acid (DNA) was extracted from the fecal material as per instructions of the NucleoSpin Soil Kit (Macherey-Nagel, Germany). This includes a bead-beating step to mechanically lyse bacterial cells during two 3-minute pulses with ceramic beads using the SpeedMill PLUS (Analytik Jena, Germany). After centrifugation, the supernatant was transferred to new collection tubes just prior to precipitation. We followed the protocol for the remaining steps. We included ten extraction blanks and six standardized communities (ZymoBIOMICS Microbial Community DNA Standard, Zymo Research, Germany).

We amplified the V4 region of the 16S rRNA gene using the 515F-806R primer pair (fwd: 5′-GTGCCAGCMGCCGCGGTAA-3′; rvs: 5′- GGACTACHVGGGTWTCTAAT-3′) [59] and added Illumina adaptor sequences using the Fluidigm Access Array for Illumina Sequencing (Access Array System for Illumina Sequencing Systems, © Standard Bio Tools, USA). Post purification (NucleoMag NGS clean-up and size select, Macherey-Nagel, Germany) and quantification (QuantiFluor dsDNA System, Promega, USA) the normalized pooled sample library was sequenced as paired-end run on the Illumina MiSeq platform at the Institute for Human Genetics, University Hospital of Bonn. A total of nine PCR controls were included.

The reads were processed with the DADA2 plug-in in QIIME 2 (v2021.8.0, [60]), removing primers, denoising reads, detecting and removing chimeras, merging paired-end reads, and differentiating between single amino acid sequence variants (ASVs, [61]). ASVs were then assigned their taxonomy using SILVA (v138) as reference database [62]. ASVs unassigned at the phylum level or identified to originate from chloroplast or mitochondrial sequences were excluded from subsequent analyses. An unrooted phylogenetic tree was build using Mafft [63] and FastTree [64]. The tree was rooted in Dendroscope [65] using an archaeon sequence (accession number: KU656649) as the outgroup, which was later removed. Metadata, including each sample’s sex, age, location, infection status, the CT-value of the respective infection, and sampling period, the taxonomy and ASV counts, and the phylogenetic tree, were imported into the Rstudio interface of R (v4.3.2, [66]) using the “phyloseq” package (v1.46.0, [67]). All sample processing and statistical analyses were performed in Rstudio.

We first confirmed the community composition of the six standardized microbial community references, which showed no large deviation in amplification across the extraction runs from the expected community composition (Supplementary Fig. 1). Next, we pruned unassigned ASVs at the phylum level, ASVs with fewer than ten reads and phyla with <0.3% total reads across all samples leaving us with 4 852 ASVs from an original of 10 975. Employing the prevalence-based contamination identification functions from the “decontam” R package (v1.16) with the default P^*^-threshold set to 0.1 [68], we identified a possible 163 taxa (3.3%) from the 10 extraction blanks and 165 taxa (3.4%) from the 9 PCR blanks. These taxa were removed. Finally, the decontaminated phyloseq object contained 4 531 taxa compiled from 7 161 284 reads. We plotted alpha-diversity rarefaction curves for each sample with the rarecurve function from the “vegan” R package (v2.6–4, [69]). Based on the curves (Supplementary Fig. 2), two fecal samples below a sequencing depth of 5 000 reads were eliminated from downstream analyses (including all controls) and one samples missing age information. Hence, a total of 218 high-quality fecal samples with on average 32 849 reads (±11 198 standard deviation; range: 8 815–62 295 reads) remained.

#### Statistical analysis

Log-transformed body condition was compared using a linear mixed effect model including sampling location, host sex and infection status on 591 adult *H. caffer D*, while accounting for capture period as random effect. *Post hoc* pairwise comparisons were performed with the “lmerTest” package (v.3.1–3, [70]). The analysis was repeated for body mass, which yielded similar results [71].

Alpha-diversity metrics (i.e., Observed ASVs, Shannon, and Faith’s Phylogenetic diversity) were calculated employing the “phyloseq” (v1.46.0, [67]) and “picante” package (v1.8.2, [72]) for samples rarefied to the sequencing depth of the sample with the lowest reads (i.e., 8 815 reads, Supplementary Fig. 2, [73, 74]). While observed ASVs measures actual bacterial ASV richness, Shannon diversity index also accounts for evenness, and Faith’s Phylogenetic diversity considers abundance and phylogenetic proximity between ASVs. The effect of sampling locations, host sex, host age and CoV infection status on each metric was then estimated using a linear mixed effect model with capture period as a random effect. The observed ASV richness was log-transformed, and Shannon diversity index and Faith’s Phylogenetic Diversity were square-rooted to meet the normality assumption. Subsequently, we assessed whether each alpha-diversity index correlated with infection intensity (measured as CT-value).

To assess inter-sample differences, we first calculated unweighted and weighted Unifrac-distances as microbial beta-diversity indices on the rarefied data agglomerated to genus level. Both distances take phylogenetic distance between ASVs into account, but whereas weighted Unifrac considers reads as proxy for ASV abundance, and, thus, represents the structure of a microbial community, unweighted Unifrac treats ASVs as either absent or present and, hence, epitomizes the composition of a microbial community. We used each index separately as response variable in a permutational multivariate analysis of variance to determine changes in centroid position associated with sampling location, host sex, host age, and CoV infection status, and PERMUtest to assess differences in distance to centroid. Following from this, we extracted the unweighted and weighted distances as matrices from all infected samples and calculated the average distance to other samples in the same infection category. The average distance denotes how dissimilar a particular sample is from others. Finally, we correlated the average distance between samples with infection intensity (measured as CT-value).

In order to understand which bacterial genera differed between the infection status, we used joint-species distribution modeling. Compared with traditional abundance-based analyses (e.g., ANCOM), joint species distribution models incorporate correlations between bacterial taxa when predicting their abundance with respect to the explanatory variable [75]. Since taxonomic databases remain biased toward identifying microbial taxa from common model organisms, the taxonomic resolution for wildlife gut microbiota at the species level is lacking [76, 77]. To avoid spurious results, we agglomerated reads at the genus level and restricted our analysis to genera with a prevalence >50% (i.e., common core [78]). The abundance was centered log-transformed (clr, [79]). Finally, we constructed a generalized linear latent variable model (GLLVM) using the “gllvm” package [75]. We evaluated multivariate microbial abundance data using the joint model with the same model structure as for testing alpha- and beta-diversity, meaning that sampling location, host age, sex and infection status were kept as main explanatory variables and capture period was used as random effect. Additionally, we accounted for sequencing depth and specified a negative binomial distribution for our response variable.

For those genera that were identified to occur more or less frequent in samples from bats infected with *Hibecovirus* CoV-2B, we wanted to ascertain whether their abundance was linked to infection intensity when compared to uninfected bats. In other words, we aimed to understand whether there is a linear or non-linear relationship with CT-value. We constructed generalized additive models with the clr-transformed abundances (i.e., reads) as response and sampling location, sex and age as categorical explanatory variables. The *Hibecovirus* 2B CT-value was included as smooth term. Sequencing depth was included as smooth term to control for differences in sequencing performance between samples. Model fit was assessed using gam.check() function of the “mgcv” package [80] and we visualized the model results using the plot_smooths() function from the “tidymv” package [81].

## Results

### Body condition

Only individuals infected with the *Hibecovirus* CoV-2B had a lower body condition than uninfected bats and those infected with the *Duvinacovirus* CoV-229E-like (Table 1). Bats infected with the other *Hibecovirus* CoV-2Bbasal show a slight tendency for a lower body condition than uninfected bats (Table 1c). Sex did not influence on body condition, the highest body condition was found in the cave Forikrom.

**Table 1:** Summary data, model results and pairwise comparisons of body condition (i.e., mass/forearm length; log-transformed) between cave location, sex and infection status in adult *Hipposideros caffer D.*

a) Body condition		n	BCI	standard error
uninfected		104	0.218	0.002
CoV-2B infected		257	0.213	0.002
CoV-2Bbasal infected		130	0.215	0.001
CoV-229E-like infected		100	0.215	0.002
**b) Final model**		**dfs**	**F-value**	**p-value**
~ infection status		3, 588	3.26	**0.021**
~ location		4, 588	8.92	**<0.001**
~ sex		1, 582	2.14	0.144
**c) Pairwise comparison**	**Estimate**	**lower**	**upper**	**p-value**
uninfected vs 2B	0.030	0.008	0.052	**0.008**
uninfected vs 2Bbasal	0.024	−0.002	0.049	0.066
uninfected vs 229E	0.006	−0.020	0.032	0.648
2B vs. 2Bbasal	−0.006	−0.026	0.014	0.534
2B vs. 229E	−0.024	−0.045	−0.002	**0.030**
2Bbasal vs. 229E	−0.017	−0.041	0.006	1.450

Significant results are indicated in bold.

### Gut microbial community composition

**Figure 1:**
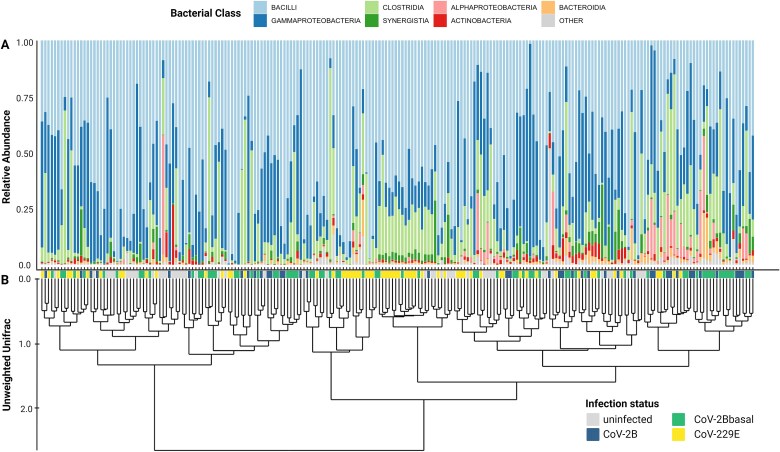
Microbiome composition and hierarchical clustering. A) The gut microbial composition of the 218 fecal samples from *Hipposideros caffer D* with known infection status B) loosely clustered by their (unrarefied) unweighted Unifrac distance-based dissimilarity. Infection status given as colored tiles (gray = uninfected; green = CoV-2Bbasal infected; blue = CoV-2B infected; yellow = CoV-229E infected).

The gut microbial community was dominated by *Bacillota* (67.0%; formerly known as *Firmicutes*) represented mainly by members of bacterial class *Bacilli*, and *Pseudomonadota* (27.2%; formerly known as *Proteobacteria*) by and large ascribed to *Gammaproteobacteria* (Fig. 1A). The 28 common core genera (i.e., found in >50% of samples) made up 86.2% of reads per sample (±15.3% standard deviation) with 71.2% reads belonging to *Lactococcus*, *Streptococcus*, *Enterococcus*, *Gemella* and *Paeniclostridium*. Hierarchical clustering showed a weak similarity in the unrarefied gut microbial composition of samples with a shared infection status (e.g., CoV-229E infected bats; Fig. 1B).

### Alpha- and beta-diversity

**Figure 2:**
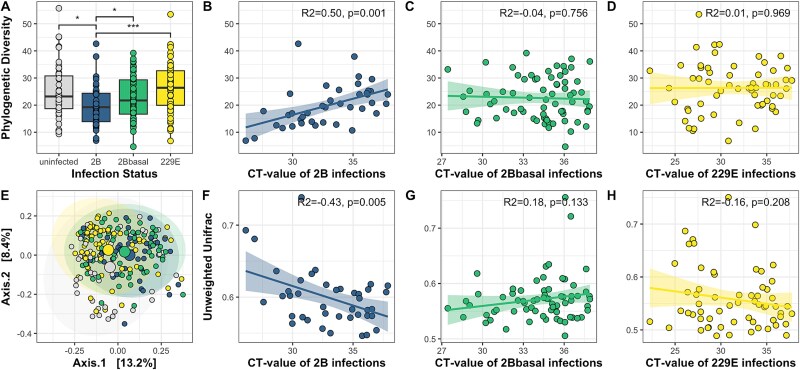
Gut microbial alpha- and beta-diversity in relation to infection status and intensity. A) Differences in gut microbial alpha-diversity (i.e., Faith’s phylogenetic diversity) based on infection status, and B–D) correlational link between alpha diversity and infection intensity of either CoV infection. E) Grouping of gut microbial composition based on beta-diversity (i.e., rarefied unweighted Unifrac distances calculated from reads agglomerated to bacterial genus), and F–H) correlational link between beta-diversity and infection intensity of either CoV infection. Asterisks indicate level of significance: ^*^ < 0.05, ^*^^*^ < 0.01, ^*^^*^^*^ <0.001. Colors reflect infection status (gray = uninfected; green = CoV-2Bbasal infected; blue = CoV-2B infected; yellow = CoV-229E infected) and larger points depict respective group centroids.

When comparing (rarefied) gut microbial alpha-diversity, infection status appeared as the single best explanatory variable (Supplementary Table 1). Post-hoc testing confirmed our prediction in that infections with *Hibecovirus* CoV-2B altered the gut microbial diversity (Fig. 2A; Supplementary Fig. 3A and B): microbial diversity was lower for bats infected with CoV-2B than for uninfected, *Hibecovirus* CoV-2Bbasal, or *Duvinacovirus* CoV-229E-like-infected bats, irrespective of the alpha-diversity index at hand (F_3,214_ = 4.40, *P* = .005; Supplementary Table 1 for observed ASVs and Shannon Diversity Index). This prompted us to test whether recorded rt-PCR CT values—a proxy for infection intensity—altered alpha-diversity. Indeed, the CT-values of bats infected with *Hibecovirus* CoV-2B were positively correlated with alpha-diversity (Fig. 2B). In other words, alpha-diversity declined with infection intensity. However, there was no change observed in alpha-diversity with infection intensity for infections caused by the other two CoVs (Fig. 2C and D).

In addition, the centroids of the (rarefied) gut microbial beta-diversity in bats infected with CoVs shifted away from the centroid of uninfected bats analyzed (Fig. 2E; unweighted Unifrac measuring composition: R2 = 0.043, F = 3.32, *P* = .001; weighted Unifrac measuring structure: R2 = 0.026, F = 2.04, *P* = .028; Supplementary Fig. 4). The unweighted Unifrac distances calculated from samples of *Duvinacovirus* CoV-229E-like infected bats were on average less dispersed than uninfected and *Hibecovirus* CoV-2B infected bats, and *Hibecovirus* 2Bbasal infected bats were less dispersed than uninfected bats (Supplementary Fig. 4). Samples did not differ in dispersion when beta-diversity was calculated as weighted Unifrac distances (Supplementary Fig. 4). Host sex and age had no effect on the gut microbial composition and structure, whereas sampling location explained some variation in unweighted Unifrac dissimilarity between samples albeit less than infection status (Supplementary Table 2). Substituting the categorical infection term once more with the continuous CT-value, we discovered that unweighted Unifrac distances negatively correlated with increased infection intensity only in *Hibecovirus* CoV-2B infected bats (Fig. 2F–H), but had no effect on weighted Unifrac distances (Supplementary Table 3).

### Joint-species distribution modeling and generalized additive models

**Figure 3:**
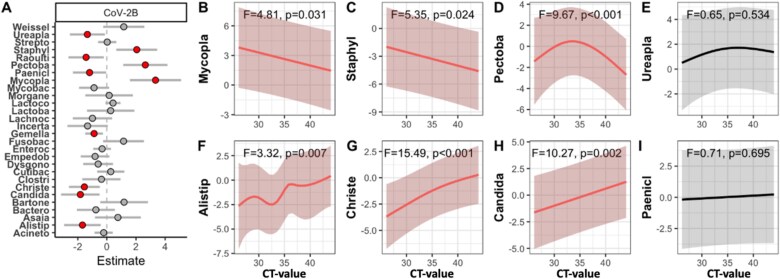
Variation in specific taxa abundance in relation to CoV infections. A) Generalized linear latent variable model indicating which common gut bacterial genera (prevalence >0.5) are found significantly (^*^P < .05, dots colored red) more or less common in CoV-2B infected *H. caffer D* than in uninfected bats, and B–I) model estimates from generalized additive models demonstrating significant (in red) relationships between the abundance of bacterial genera and infection intensity. Abbreviations: Weissel = *Weissella*; Ureapla = *Ureaplasma*; Strepto = *streptococcus*; Staphyl = *staphylococcus*; Raoulti = *Raoultibacter*; Pectoba = *Pectobacterium*; Paenicl = *Peniclostridium*; Mycopla = *mycoplasma*; Mycobac = *mycobacterium*; Morgane = *Morganella*; Lactoco = *Lactococcus*; Lactoba = *lactobacillus*; Lachnoc = *Lachnoclostridium*; Incerta = *Incertae Sedis*; Fusobac = *Fusobacterium*; Enteroc = *enterococcus*; Dysgono = *Dysgonomonas*;; Clostri = *Clostridium sensu stricto 1*; Christe = *Christensenellaceae R-7 group*; Candida = *Candidatus Soleaferrea*; Bartone = *Bartonella*; Bactero = *Bacteroides*; Alistip = *Alistipes*; Acineto = *Acinetobacter*.

For a more detailed understanding about which gut bacterial members varied between infections, we then employed a joint species distribution model on the most common bacterial genera. Of the 28 core genera, ten varied significantly between uninfected and CoV-2B infected bats (Fig. 3A; Supplementary Table 4). Among others, bacteria of the genus *Mycoplasma* and *Staphylococcus* were more abundant in the gut microbial community of CoV-2B infected bats. *Christensenellaceae* R-7 group and *Alistipes* were less frequently detected in infected bats. By contrast, fewer genera were associated with the other two CoV infections (Supplementary Table 4). Noteworthy is that the abundance of *Gemella* declined in all infected bats. Few genera differed between locations, or among the sexes, whereas several genera were found more or less abundant in subadults than in adults (Supplementary Fig. 5).

We predicted that several of these genera either decrease or increase with CoV-2B infection intensity, and constructed generalized additive models to test for linear and non-linear relationships with the CT-values. As expected, *Mycoplasma* and *Staphylococcus* were most frequent in samples from bats with the lowest CT-values, and declined linearly in abundance in relation to uninfected bats (Fig. 3B and C). Vice versa, members of the genera *Christensenellaceae* R-7 group, *Alistipes*, and *Candidatus Solearferrea*, by comparison, were less prevalent at low CT-values, when compared to uninfected bats (Fig. 3F–H; Supplementary Table 5). Other genera, including the fourth and fifth most common core genera *Gemella* and *Paeniclostridium*, showed no significant linear change with infection intensity (Fig. 3E and I, Supplementary Table 5).

## Discussion

Bats host a variety of viruses, but few records of health repercussions exist [18, 82, 83]. We tested whether infections with any one of three bat CoVs altered body condition and changed the gut microbial community in wild *H. caffer D*, and predicted to observe the most prominent responses to infection with the *Hibecovirus* CoV-2B, to which this bat species seems susceptible [51, 53]. Our observations yielded three insights: (i) the impact on body condition and the gut microbial community seems to be virus dependent with CoV-2B infections linked to reduced body condition and microbial diversity while the other *Hibecovirus* CoV-2Bbasal and the *Duvinacovirus* CoV-229E-like had no detectable effect; (ii) the gut bacterial diversity declined and the community composition became more dissimilar among the more severely CoV-2B infected bats; and (iii) the gut microbial community of CoV-2B infected bats was enriched with potentially pathogenic bacterial genera and depauperated of health-associated taxa. These findings showcase how pathogen reservoirs, and specifically bats respond to virus infections but also underscore that responses are likely specific to the co-evolutionary relationship between host and virus.

*H. caffer D* infected with the distantly SARS-related *Hibecovirus* CoV-2B exhibited a lower body condition, a reduced gut microbial diversity, and a shift in gut microbial composition. A reduction in body condition was also seen in Hendra virus-positive black flying foxes (*Pteropus alecto* [19]) and CoV-positive Lyle’s flying foxes (*P. lylei* [23]). This weight loss may not actually be caused by the infection but rather due to an increased probability of infection during periods of seasonal weight loss in flying foxes [24]. Seasonality in CoV shedding is also known from some studies but absent in others (e.g., [84, 85]). Seasonal variation in insect prey could similarly explain changes in the gut microbiota [86]. However, we accounted for sample period statistically and would otherwise expect to see similar results for individuals infected with any of the other two CoVs, which tend to have overlapping periods of high prevalence [51]. This is not the case. Infections with the most recent ancestor to the HCoV-229E and the more basal *Hibecovirus* demonstrated no apparent differences in body condition or gut microbial community. Host responses to infection are therefore likely virus-dependent. Furthermore, gut dysbiosis may not even translate into changes in body condition. For example, in Astrovirus infected Jamaican fruit bats body condition remained unaffected in spite of a reshuffling of the taxonomic gut microbial profile [41]. This raises the possibility that gut microbial dysbiosis is an invisible and often overlooked health marker in epidemiological studies on bats.

Unlike the previous work on the impact of Astrovirus infections on the gut microbial community [41], we demonstrated that alpha- and beta-diversity correlated with infection intensity. These findings imply a quantifiable dimension to the Anna Karenina Principle [7] where viral infection intensity governs the level of gut microbial idiosyncrasy. During the latent infection with the simian immunodeficiency virus, chimpanzees also show little to no differences in their gut microbiota, but the gut microbial composition was severely altered in apes dying from an acquired immunodeficiency syndrome-like immunopathology [11]. The gut microbial community of patients with hepatitis C also progressively changes with disease severity [87], and hamsters and macaques clinically infected with SARS-CoV-2 showed trends toward higher gut microbial dissimilarity over the course of the disease [45, 46]. Thus, our finding that microbial diversity and dissimilarity scales with infection intensity is likely. Nevertheless, without repeated samples from the same bat we cannot be certain at which time point of the infection (i.e., pre-, post-, or during CT-peak) the sample was taken [26], and the observation remains correlational.

The gut microbial community consisted largely of *Bacillota* and *Pseudomonadota*, closely matching the composition of other insectivorous bats [29, 35, 88]. Yet, many of the 28 core genera varied in abundances depending on infection status. Several presumably beneficial bacteria belonging to the order of *Bacteroidales* and *Clostridiales* declined in abundance in CoV-2B infected bats. Members of these bacterial orders were depleted in patients with more severe Covid-19 symptoms compared to patients presenting only mild symptoms [89]. The abundance of several members of the *Christensenellaceae* R-7 group was also negatively correlated with SARS-CoV-2 viral load and inflammatory markers in macaques [46]. At the same time, potentially pathogenic bacteria became enriched in CoV-2B infected bats. The genus *Mycoplasma*, which features bacterial species pathogenic to humans (e.g., *Mycoplasma pneumoniae*), livestock (e.g., *Mycoplasma bovis*), and wild animals (e.g., *Mycoplasma ovipneumoniae* [90]), was more abundant in CoV-2B positive bats and increased linearly with infection intensity. *Mycoplasma* was also common in Jamaican fruit bats infected with an Astrovirus [41, 91]. An open question remains whether such taxonomic changes render the gut microbial community functionally incapacitated, which is ideally addressed with multi-omics.

Our line of argument rests on the assumption that a more dysbiotic state, marked by a loss of overall bacterial diversity, increased idiosyncrasy, and an enrichment of potentially pathogenic members at the expense of beneficial bacteria, is a consequence of the *Hibecovirus*-2B infection. Equally feasible is that stress altered the gut microbial diversity initially thereby diminishing the host's ability to withstand subsequent colonization by *Hibecovirus*-2B and increasing susceptibility to the virus (e.g., nutritional stress [92, 93], asynchronous co-infections [58] and human disturbance [94]). This explanation is challenged by the finding that infections with the *Duvinacovirus* and *Hibecovirus* 2Bbasal reach similar intensities, regardless of variations in the host’s gut microbial diversity. Without repeated sampling from bats as they progress through the infection and a functional profile of gut symbionts, we are unable to test whether the observed changes in the taxonomic memberships are due to the infection, or even whether they have ramifications for microbiome-mediated metabolic and immunological functions [17, 26, 95], and ultimately, host fitness [5]. Ideally, future studies could non-invasively probe for physiological markers that play a role in mediating host immunity and communicating with gut bacteria (e.g., short chain fatty acids, IgA/IgG, mucin [96, 97]), and new multi-omics approaches may provide a higher resolution of the diversity of pathogens infecting bats and other hosts [98, 99].

Taken together, our results demonstrate a link between the *Hibecovirus* CoV-2B infection and changes in the gut microbial community of a putative virus reservoir. We provide evidence that the gut bacterial diversity declined and the community composition became more dissimilar among the more severely infected bats in line with expectations based on the Anna Karenina principle [7]. Furthermore, potentially pathogenic bacteria took hold, while common symbionts declined in the depauperated gut microbial community of acutely infected bats. If gut dysbiosis was a consequence of more severe infections, as we propose here, this might lead to poorer coverage of bacterial services hosts rely upon [13]. In turn, reservoir species, such as bats, may still suffer from viral infections via a phenotypically invisible health indicator—the microbiota [3, 31].

## Supplementary Material

SupplementaryFigures_311024_ycae154

SupplementaryTable1_ycae154

SupplementaryTable2_ycae154

SupplementaryTable3_ycae154

SupplementaryTable4_ycae154

SupplementaryTable5_ycae154

## Data Availability

Metagenome data can be accessed from NCBI under BioProject no. PRJNA1096136. Meta data can be accessed from github (https://github.com/DominikWSchmid/GhanaHippos_CoV_microbiome).

## Code availability

Workflow and code are freely accessible from github (https://github.com/DominikWSchmid/GhanaHippos_CoV_microbiome).
