# Supplementary material for: Hibecovirus (genus Betacoronavirus) infection linked to gut microbial dysbiosis in bats
Source: ISME Commun. 2024 Dec 16;5(1):ycae154. doi: 10.1093/ismeco/ycae154 (PMC11936109; doi:10.1093/ismeco/ycae154)
Supplement: SupplementaryFigures_311024_ycae154 [file supplementaryfigures_311024_ycae154.docx]

**Supplementary Information**

***Hibecovirus* (genus *Betacoronavirus*) infection linked to gut microbial dysbiosis in bats**

Dominik W. Melville*, Magdalena Meyer*, Alice Risely, Kerstin Wilhelm, Heather J. Baldwin, Ebenezer K. Badu, Evans Ewald Nkrumah, Samuel Kingsley Oppong, Nina Schwensow, Marco Tschapka, Peter Vallo, Victor M. Corman, Christian Drosten, Simone Sommer


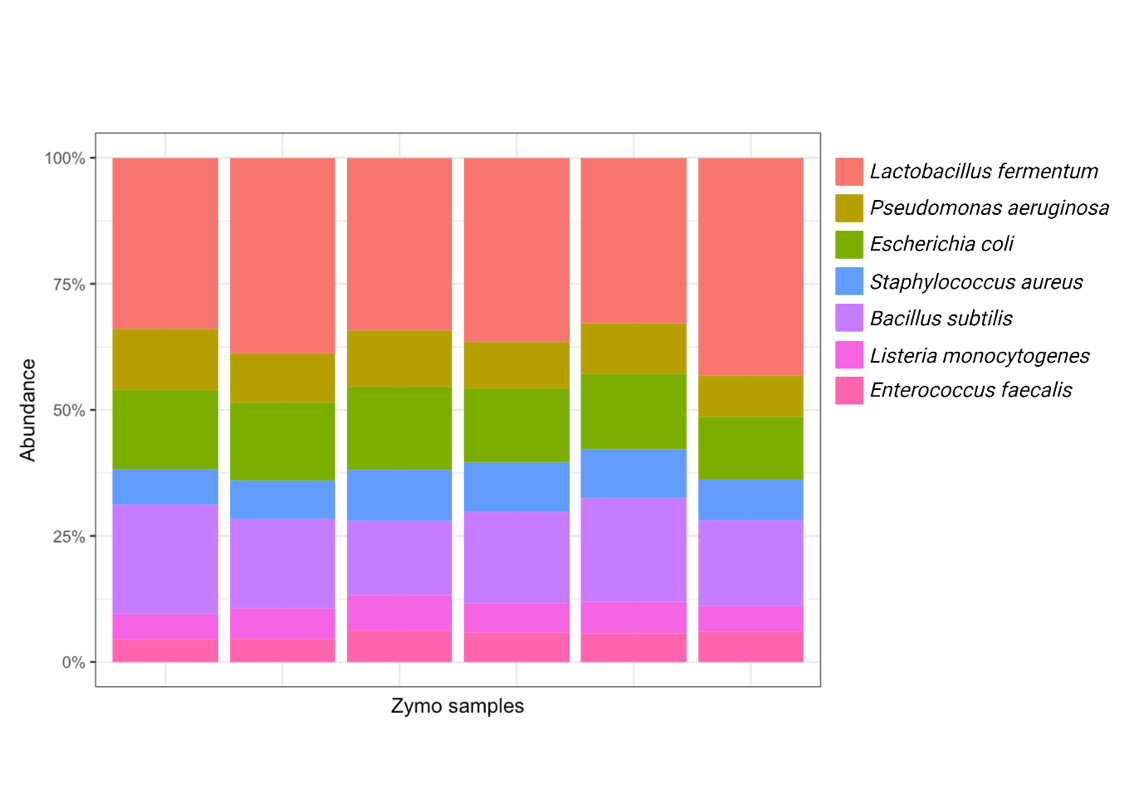


**Supplementary Figure 1. Consistency in extraction and sequencing protocols.** A total of six ZymoBIOMICS microbial community standards were included to assess consistency in extraction and sequencing protocols.


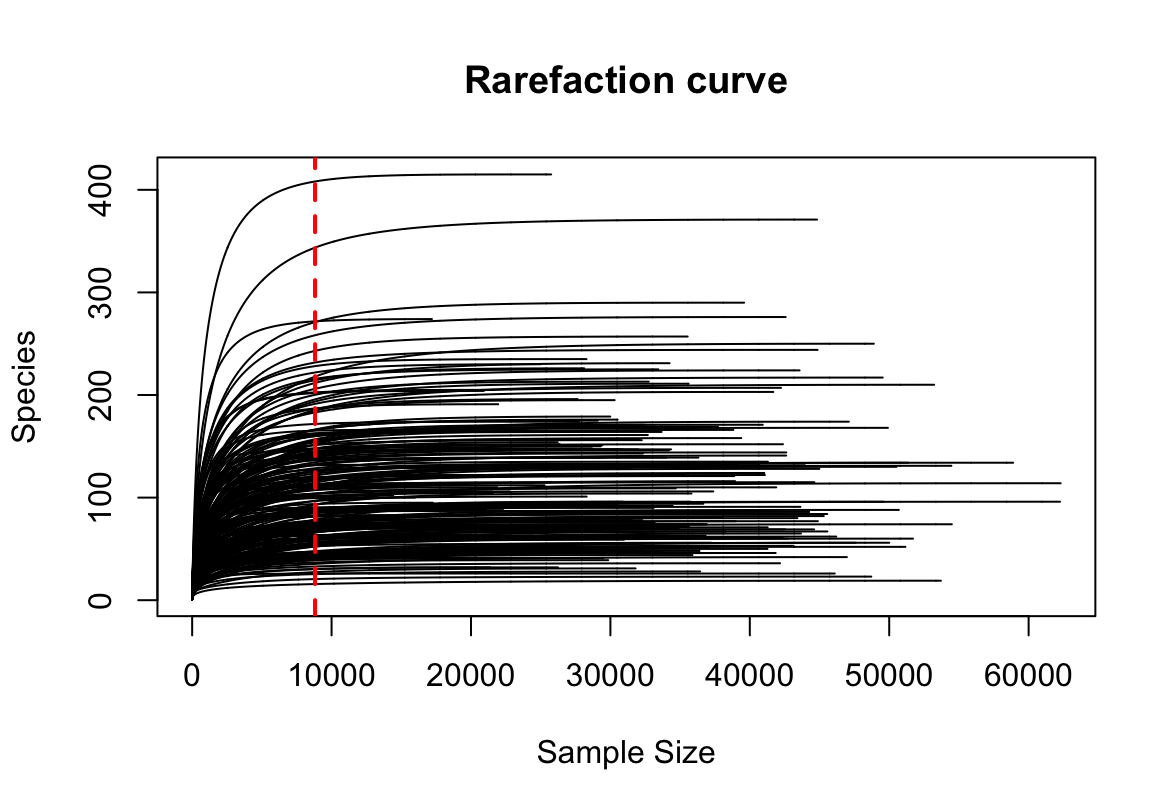


**Supplementary Figure 2. Rarefaction curve for all samples.** Red dashed line indicates read count of the sample with the lowest number of reads (i.e., 8815) and, hence, forms the rarefying threshold.


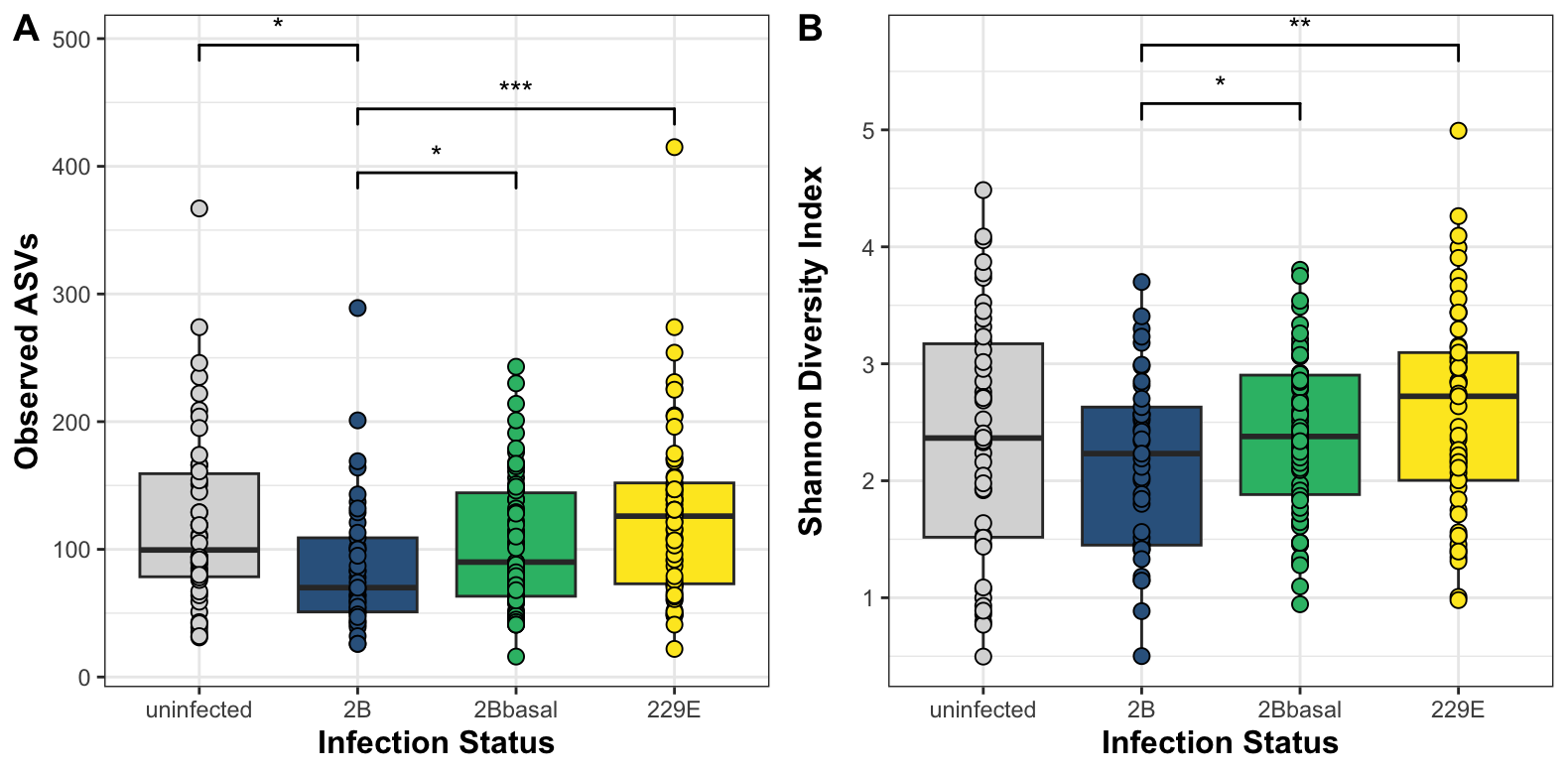


**Supplementary Figure 3. Differences in gut microbial alpha-diversity measured as A) Observed ASVs and B) Shannon Diversity Index in relation to infection status.** Asterix indicate level of significance: * < 0.05, ** < 0.01, *** <0.001.


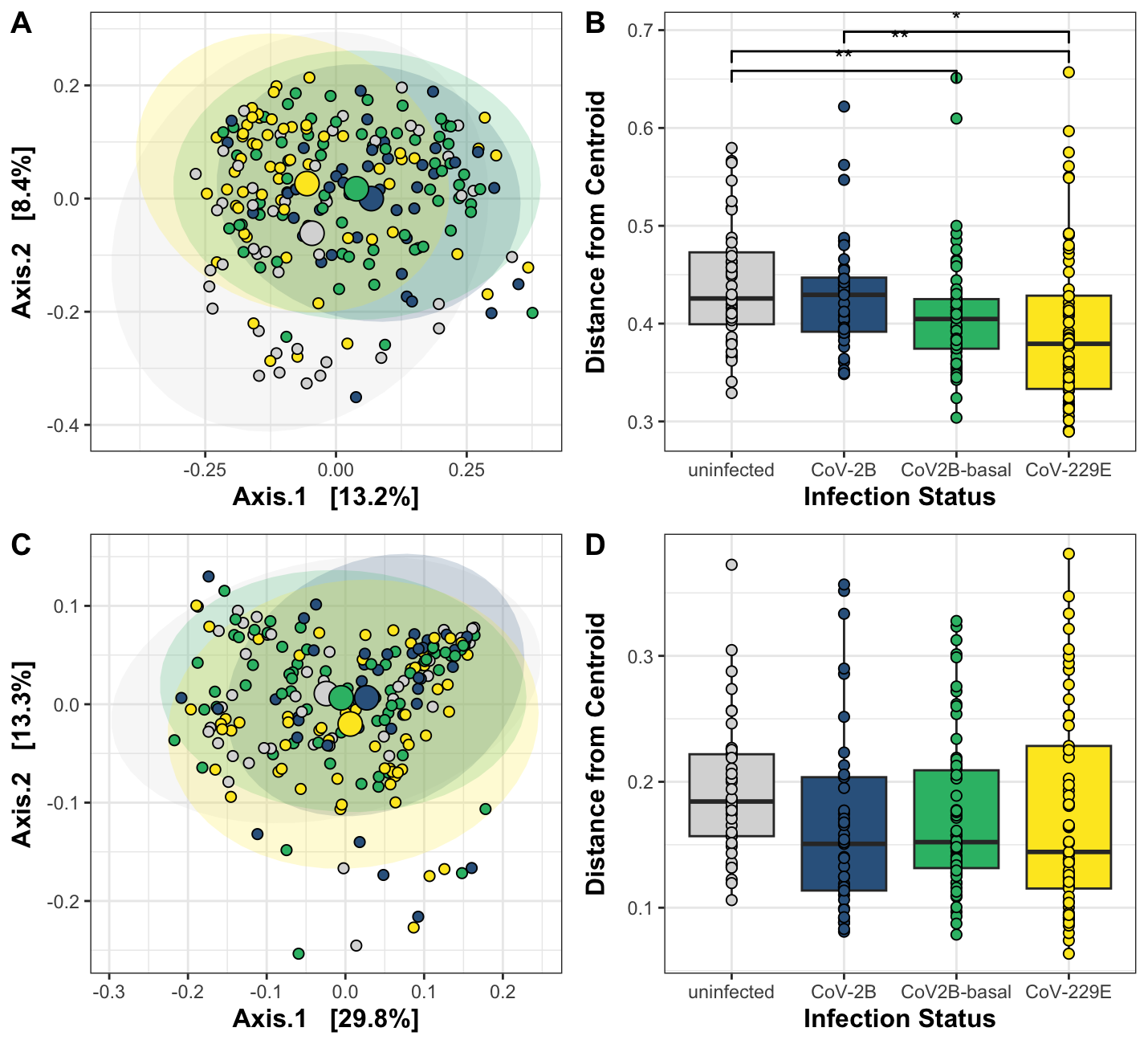


**Supplementary Figure 4. Differences in gut microbial beta-diversity in relation to infection status. A-B) Unweighted and C-D) weighted Unifrac distances depicted in a PCoA and as distances from centroid.** Ellipses are drawn using 95% confidence intervals. Larger filled points represent group centroids. Asterix indicate level of significance: * < 0.05, ** < 0.01, *** <0.001.


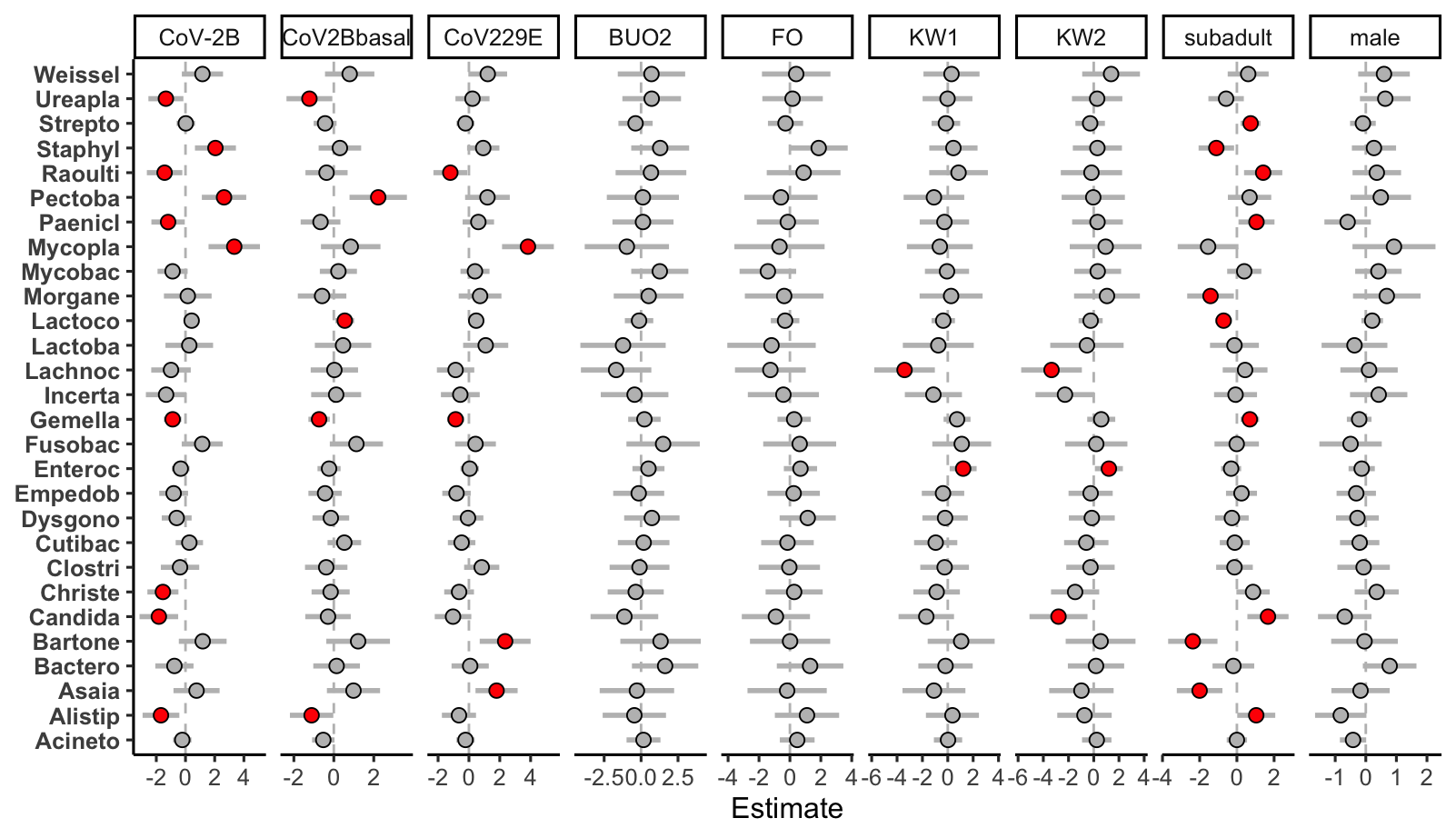


**Supplementary Figure 5. Generalized linear latent variable model indicating which common gut bacterial genera (prevalence > 0.5) are found significantly (dots colored red) more or less common in *H. caffer* *D* with an infection (CoV-2B, CoV-2Bbasal or CoV-229E) or between cave sites, age classes and sexes (compared with the indicated reference).** Abbreviations: Weissel=*Weissella*; Ureapla=*Ureaplasma*; Strepto=*Streptococcus*; Staphyl=*Staphylococcus*; Raoulti=*Raoultibacter*; Pectoba= *Pectobacterium*; Paenicl=*Peniclostridium*; Mycopla=*Mycoplasma*; Mycobac=*Mycobacterium*; Morgane=*Morganella*; Lactoco=*Lactococcus*; Lactoba=*Lactobacillus*; Lachnoc=*Lachnoclostridium*; Incerta=*Incertae Sedis*; Fusobac=*Fusobacterium*; Enteroc=*Enterococcus*; Dysgono=*Dysgonomonas*;; Clostri=*Clostridium sensu stricto 1*; Christe=*Christensenellaceae R-7 group*; Candida = *Candidatus Soleaferrea*; Bartone=*Bartonella*; Bactero=*Bacteroides*; Alistip=*Alistipes*; Acineto=*Acinetobacter*.
